# Royal Westminster Ophthalmic Hospital

**Published:** 1829-12

**Authors:** 


### ROYAL WESTMINSTER OPHTHALMIC HOSPITAL.

### *Report of some particular Cases, treated by different Methods, at the Royal Westminster Ophthalmic Hospital.*

*To the Editor of the London Medical and Physical Journal.*

Sir: You were pleased to express a desire to have the particulars of the first of the following cases sent to you, on seeing her the day she presented herself, (October 27th)

conceiving, as you then said, that it was next to impossible her eyes could be saved. I have added, by desire of Mr. Guthrie, five others, illustrative of different modes of treating similar diseases; and have the honour to be, sir,

Your obedient servant,

J. R. Taylor, *House-surgeon.*

*November*, 1829.

Case I. Catherine Bulkley, æt. thirty, applied October 27th, under the following circumstances: Has been subject to lippitudo for several years; a month ago was attacked by inflammation of the ball of the right eye, accompanied by swelling of the lids, pain, and great discharge. These symptoms continued for about a fortnight, when the inflammation subsided for five or six days, the discharge continuing; the inflammation then returned, and has been since increasing in violence. A week ago the left eye also became inflamed. She has had a gonorrhœa for more than a month. Both eyes are greatly inflamed; there is great chemosis of the ball, encroaching upon the edge of the cornea, together with superficial ulceration, and some degree of loss of transparency of that part; considerable purulent discharge; great pain in her head and eye; discharge scalding hot.

The nitrate of silver ointment was immediately applied to both eyes by Mr. Guthrie himself, in order, to ensure its proper application in a case of so much importance to the patient, whose eyes were in the utmost danger. He also directed her to be cupped to eight ounces on each temple. No internal medicine to be given; but to attend at his house at ten o'clock the next morning.

28th.—The disease is arrested; she is something better in every respect.

29th.—Can open her eyes and see her way, and says that she is considerably better. The ointment applied, and to be cupped to twelve ounces.

31st.—Continues improving: chemosis, pain, and purulent discharge, considerably less. Having no settled home nor means of subsistence, Mr. Guthrie sent her to-day to the Westminster Hospital.—The ointment applied.

November 2d.—Better. Ointment repeated.

7th.—The chemosis has disappeared; eyes considerably better, but she complains of pain in the left side of her head, and is ordered to be cupped to eight ounces behind the ear.—Haustus Inf. Sennæ cum Sulph. Magn.

9th.—Discharge ceased, and the inflammation of the ball of the eye nearly gone; says that her headach is not better, and thinks it arises from want of more food.—Ordered half diet. Rep. Ung.

11th.—Says that the half diet has cured her headach, and is supposed to be afraid of being discharged the hospital.—Infus. Sennæ omni mane.

16th.—Ordered to be discharged at the end of the week, and to take some of the Ung. Hydrarg. Nitr. dil. to apply to the edges of the lids for the lippitudinous affection.

Case kept by Mr. James.

Case II. Wm. Smith, æt. fifty, admitted August 25th, 1829. Has been attending at the hospital since the above date, for an amaurotic affection of his right eye. Three days ago, however, this eye was attacked by inflammation, accompanied with severe pain.

November 14th.—The conjunctiva is now very much inflamed, and there is very great chemosis; so much so, that the cornea is partly concealed. Lids are also inflamed, and very much swollen; and there is a great discharge of water from the eye. He complains of pain in his forehead and eye.—Cucurb. cum ferro ad ℥x. Pulv. Jalapæ comp. ʒi. statim.

17th.—Still complains of pain; chemosis and great swelling of the lids not much diminished.—Eighteen ounces of blood to be taken immediately from the temporal artery. The Ung. Argenti Nitratis to be applied forthwith. Emplast. Canth. pone aurem hora somni. Capiat Calomel gr. vi. nocte. Magn. Sulph. ℥i. mane.

18th.—Says he felt pain for half an hour after the ointment was applied, but is much better this morning.—Warm fomentation.

19th.—Improving.

21st.—Is much better; chemosis subsiding fast. The eye is out of danger.—Repeat the ointment.

Case kept by Mr. Richards.

Case III. Mary Ann Price, æt. fourteen, admitted November 10th, 1829. Five days ago she began to feel pain in her left eye, extending to the temple, accompanied by increased lachrymation. There is acute inflammation of the conjunctiva, with great chemosis; cornea clear; iris healthy. Pulse quick, skin hot, tongue furred, bowels confined.—To be cupped to ℥viij. Appl. Ung. Arg. Nitr. Pulv. Jalapæ c. ʒss.

12th.—Says that her eye was easier yesterday than it is to-day. The inflammation and chemosis appear much the same as on the 10th. Bowels have not been opened.—Rep. pulv. et Unguent. Arg. Nitr.

14th.—The chemosis a little diminished.—Rep. Unguent. Pil. Hydr. cum Cambog. gr. x. nocte. Magn. Sulph. ℥ss. mane.

17th.—The left eye is much better; but the right was attacked on the 15th, and is now more inflamed than the left.—The ointment of the oxymuriate of mercury to be applied to the right eye, and the nitrate of silver ointment to the left as before.

21st.—Was so much better on the 19th that she did not come to the hospital. The right eye was more benefited by the oxymuriate ointment than the left by the Ung. Arg. Nitr. Both are out of danger.—To continue the ointment to each eye respectively.

Case kept by Mr. Davis.

Case IV. James Halfacre, æt. thirteen, admitted November 13th, 1829, with ulceration of the cornea of both eyes.

The left eye became inflamed some weeks ago, and the right a few days after. He has had leeches and lotions applied, but both eyes have been gradually getting worse, and now there is a very large ulcer, nearly occupying the whole of the cornea of the right eye, but it has not yet opened into the anterior chamber; there is great inflammation of the conjunctiva of the ball and lids, which are also thickened. The right eye is not so much inflamed, and the ulcer in the cornea is smaller. He says that he suffers very little pain in his eyes, and none whatever in his head; yet he cannot bear the light without suffering great pain, and, when the lids are raised, a quantity of water runs from the eyes.—To lose six ounces of blood from the left temple, and to have the nitrate of silver ointment applied to both eyes.—Pulv. Jalapæ c. ℈i. cras mane.

15th.—Better. The inflammation in both eyes abated, and the ulcer in the left cornea does not appear to have spread.—Empl. Canth. pone aurem sinist.

17th.—The inflammation of the conjunctiva of the right eye has very much diminished; the cornea is clearer, and the ulcer healing. The left eye is also less inflamed. The blister rose very little.—Rep. Applic. Ung. Arg. Nitr. singulo oculo.

19th.—Repetatur Ung. Pergat.

21st.—Right eye nearly well, very little inflammation remaining; ulcer nearly filled up. The left eye is also better; ulcer much less, and the inflammation of the conjunctiva considerably diminished. He is now able to see distinctly with his right eye, which, from the inflammation and ulceration of the cornea, he could not do when he first came to the hospital.—Rep. unguent.

This case is still under treatment, and is one of the most remarkable of those in which the new method of treatment has been tried; the child having got regularly worse during seven weeks, until it was adopted.

Case kept by Mr. Taylor.

Case V. Emma Hay, æt. fifteen, admitted October 27th, 1829. There is an extensive ulcer of the cornea of the right eye, the whole of the cornea being opaque. Acute inflammation also of the cornea, which has a zone of red vessels surrounding it. She has been ill for three years, but of late the ulcer and opacity have much increased. She has been bled and leeched repeatedly, with very little relief, and has had various applications to the eye. Is now quite unable to distinguish any object with the right eye, and complains of intense pain in the eye and forehead. Great intolerance of light and constant lachrymation. Bowels generally confined; tongue white; pulse quick, and rather full. The sclerotic coat of the left eye is a little inflamed, and there is also an appearance of chronic inflammation of the conjunctiva of the tarsi of that eye.

October 27th.—Applic. Ung. Argent. Nitratis oculo dextro.—℞. Pulv. Jalapæ comp. ʒi. statim sumend.

29th.—The inflammation and opacity of the cornea is much less to-day, and the vascularity is much diminished. She says that, in six hours after the first application of the ointment, the pain in the eye and forehead ceased, as did also the intolerance of light. To-day there is scarcely any lachrymation. She observes that her eye has not been so easy and free from pain for the last three years as it is to-day.—Rep. Ung. Argent. Nitratis et Pulv. Jalapæ c. ʒi.

Nov. 2d.—The zone of red vessels surrounding the cornea has quite disappeared, and the ulcer and opacity are rapidly lessening. Eye perfectly free from pain and lachrymation.—Rep. Ung. Argent. Nitratis. Rep. pulv.

Nov. 5th to 18th.—Rep. unguent.

Since the last report, the opacity and ulceration of the cornea have gradually and entirely disappeared. She is now able to see perfectly well with both her eyes, and there remains no trace of disease.

Discharged, cured.

Case kept by Mr. Richards.

Case VI. Thomas Maize, æt. one year and a half, admitted November 17, 1829, with muco-purulent ophthalmia.

For the last three weeks the child has had a slight discharge of matter from both eyes, attended with external discoloration of the lids, which were also inflamed. Not much apparent inflammation of the ball. For this simple washes were used. Until yesterday morning, the disease rapidly increased in intensity, the lids being much swollen and discoloured, and entirely closing up the eyes. Discharge also much increased in quantity, and of a more yellow appearance. On separating the lips, the corneæ appeared transparent, conjunctiva of the ball being much injected and tumefied. Bowels not open.—Appl. Ung. Nigr. ad oculum dextrum. Ung. Hyd. Oxym. ad oculum sinistrum. Lotio Aluminis ter quater die utend. Pulv. Alter. i. omni nocte et mane sumend.

17th.—The child's mother states that, within an hour after the application of the ointments, it opened its eyes, and was enabled to keep them open all day. Very little inflammation remains of the ball; much more on the lids; not much discharge at present; lids are not swollen. Bowels freely open.—Rep. lotio et pulv.

19th.—Cured.

Case kept by Mr. Foote.*

[To be continued.] vol. lxiii. p. 47.

* Professor Graefe strongly recommends the application of a concentrated solution of nitrate of silver in cases of chronic ophthalmia with great purulent secretion. (*Rapport. sur l'Institut. Chir. et Ophthal. de Berlin.*)—Editors.

